# Lactoferrin: Properties and Potential Uses in the Food Industry

**DOI:** 10.3390/ijms26041404

**Published:** 2025-02-07

**Authors:** Ranya Demir, Sümeyye Sarıtaş, Mikhael Bechelany, Sercan Karav

**Affiliations:** 1 Department of Molecular Biology and Genetics, Çanakkale Onsekiz Mart University, Çanakkale 17000, Türkiye; ranyaddemir@gmail.com (R.D.); sumeyyesaritas@stu.comu.edu.tr (S.S.); 2 Institut Européen des Membranes (IEM), UMR 5635, University Montpellier, ENSCM, CNRS, F-34095 Montpellier, France; 3 Functional Materials Group, Gulf University for Science and Technology (GUST), Masjid Al Aqsa Street, Mubarak Al-Abdullah 32093, Kuwait

**Keywords:** lactoferrin, food industry, fortification, safety usage, antimicrobial, antiviral, antioxidant, anticancer, probiotic, therapeutical potential

## Abstract

Lactoferrin (LF) is an 80 kDa glycoprotein that contains approximately 700 amino acids and is a member of the transferrin family. The essential properties of LF, including antimicrobial, antiviral, anticancer, anti-inflammatory, antioxidant, and probiotic effects, have been studied for decades. The iron chelation activity of LF is significantly associated with its antimicrobial, anti-inflammatory, and antioxidant properties. Owing to its probiotic and prebiotic activity, LF also facilitates the growth of beneficial microorganisms and iron-defense immediate-effect properties on pathogens. Additionally, the ability to regulate cell signaling pathways and immune responses makes LF a prominent modulatory protein. These diverse characteristics of LF have gained interest in its therapeutic potential. Studies have suggested that LF could serve as an alternative source to antibiotics in severe infections and illnesses. LF has also gained interest in the food industry for its potential as an additive to fortify products such as yogurt, infant formula, and meat derivatives while also improving the shelf life of foods and providing antimicrobial and antioxidant activity. Prior to using LF in the food industry, the safety and toxicity of food processing are necessary to be investigated. These safety investigations are crucial for addressing potential harm or side effects and ensuring a healthy lifestyle. This review discusses the attributes and safety of LF, particularly its exploitation in the food industry.

## 1. Introduction

Lactoferrin (LF), also known as lactotransferrin or red milk protein, is found in the milk products of mammalian species [1]. It was first isolated from bovine milk in 1939 by Sorensen and Sorensen [2] and consists of about 700 amino acids, folded into two globular domains connected by an alpha helix [3,4]. LF is structurally unique, in which the characteristics of its components are derived from its presence in biological activities on infant and adult biosystems [5,6]. LF possesses beneficial effects, including antiviral, anti-inflammatory, antimicrobial, and antiparasitic activity, and also facilitates the growth of beneficial microorganisms and iron-defense immediate-effect properties on pathogens [7]. Owing to these diverse effects, LF is also known as one of the nutraceutical proteins in the body [8]. LF also functions as an immune modulator by regulating immune system activity according to the requirements of the body [5]. LF possesses affirmative effects on gut health, primarily by enhancing intestinal epithelium and providing the growth of probiotics [9,10]. Owing to its diverse properties, LF is considered one of the major proteins in milk serum. Moreover, LF is widely distributed in different mammalian species, including the secondary granules of neutrophils, bronchial and intestinal secretions, tears, and milk [11].

Colostrum is the initial milk secreted from mammary glands in postpartum in the very first days after birth [12]. The composition of colostrum is enriched with essential nutrients, such as oligosaccharides, and immune factors that are beneficial for neonates [7,13,14]. Additionally, colostrum-sourced LF concentrates aid in the immune system and its mobility functions [11,15]. This characteristic of LF is presumably associated with its health-promoting properties, including anti-inflammatory, antiviral, antibacterial, and antioxidant. The highest concentrations of LF are distributed in colostrum, containing four times more LF than those found in mature milk [7].

Bioactive ingredients of colostrum, including LF, have been analyzed in numerous clinical studies investigating their effects on coronavirus disease 2019 (COVID-19), namely, inhibiting the viral entry and hindering viral attachment to the cell [16,17]. Attributed studies indicate that the constituents of colostrum possess antiviral action and have a modulatory effect on innate/adaptive immune responses [5]. From this perspective, a recent clinical study has suggested its potential as a therapeutic agent in the treatment of Parkinson’s disease (PD). According to the results of the study, LF may exhibit a potential activity in both neuroprotection and selective immunotherapy [18]. LF possesses the ability to mitigate neurodegeneration and provide a conservation for dopaminergic cells against oxidative stress [19]. The mechanism by which LF regulates iron metabolism and storage is important to agonize against PD. In recent studies, it has also been demonstrated that LF promotes the transportation of drugs, therefore, indicating its potential as a therapeutic adjuvant to ameliorate PD [18,20]. Currently, Japanese scientists incorporate bovine lactoferrin (bLF) into various food products, including infant formula, yogurt, specialized milk-based beverages, nutritional supplements, pet foods, and cosmetics [21]. Similarly, additional countries, including Indonesia, South Korea, and Spain, strive for infant formulas enriched with bLF. However, it remains unclear whether bovine lactoferrin exhibits similar behaviors as human lactoferrin (hLF) in all proposed applications [1].

In this review article, characteristics of lactoferrin and related mechanisms stemming from its health-enhancing properties, including iron chelation activity, inducing apoptosis, and suppressing inflammatory cell activation or proliferation, are summarized. The affiliated mechanisms and therapeutic potential of LF are discussed. Moreover, its effect as a potential treatment on various diseases and some microbial infections is also discussed. LF applications in the food industry, including fortification on different additional foods, are analyzed. The effects and safety usage of LF fortification are reviewed.

## 2. Lactoferrin and Its Properties

**Figure 1:**
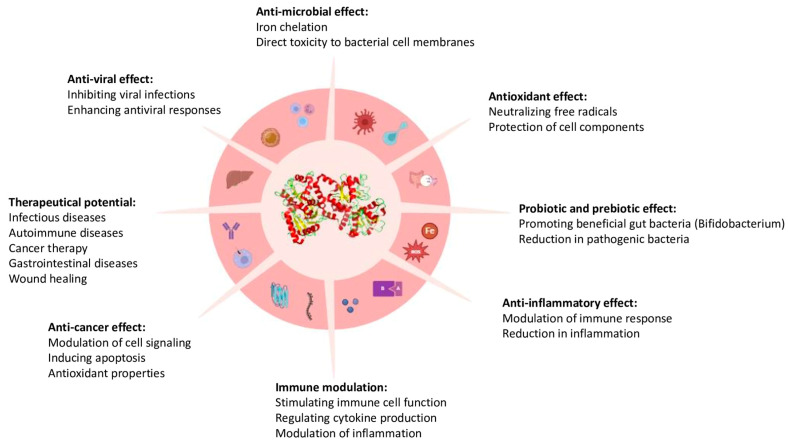
General presentation of lactoferrin and its properties.

LF transfers and conjugates Fe^3^⁺ ions and is one of the essential components for both adaptive and innate immune systems [1,22]. LF possesses various essential roles, including antimicrobial, antioxidant, antiviral, anticancer, immunomodulatory, and anti-inflammatory effects. Moreover, LF has been studied in various fields as well as from different sources and in several conditions (Figure 1) [23].

The iron-binding characteristic of LF is directly associated with its antimicrobial activity [24]. The antimicrobial activity of LF modulates defense mechanisms by terminating the growth of a wide range of pathogens, such as fungi, viruses, and bacteria [25]. The regulation role of LF in immune cells, such as neutrophils, to avoid infections has been widely demonstrated [26]. Furthermore, LF plays a crucial role in suppressing reactive oxygen species (ROS) to protect the cells from oxidative damage [27]. By acting as a growth factor, it can promote the growth and development of a variety of tissues [28]. Along with these features, the potential mechanisms of LF have been explored in multiple fields (Table 1).

### 2.1. Antimicrobial Effect 

**Figure 2:**
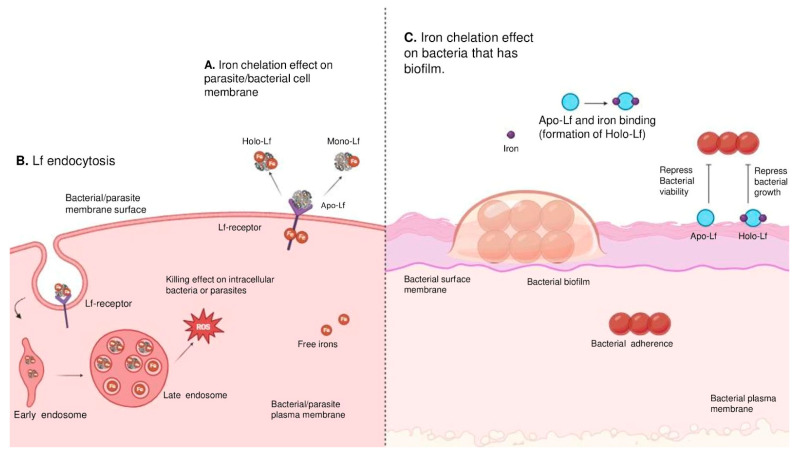
Illustration of antimicrobial effects of lactoferrin (**A**). LF chelates iron on a bacterial or parasite’s cell membrane surface to deprive an essential nutrient from the host (**B**). LF can be engulfed by parasites through endocytosis. This process helps LF to release its iron into the cytoplasm, causing oxidative stress in the form of ROS, ultimately causing cell death and proving its efficiency against intracellular parasites [70] (**C**). A model for the inhibition of biofilm and the growth regarding iron chelation of LF [72].

The iron-binding mechanism of LF plays a crucial role in the immune system [67]. By sequestering iron, LF inhibits the growth of harmful bacteria, directly damages cell walls, and induces cell death, thereby preventing bacterial infections or diseases and contributing to food preservation [26,68]. LF demonstrates a direct interaction with charged lipopolysaccharides (LPSs) of Gram-positive bacteria on account of the *N*-terminus in its structure, thereby decreasing the negative charge on the cell wall [8,26,69]. The iron chelation property of LF induces the mitigation of bacterial and parasitic growth by depriving essential nutrients for their growth (Figure 2A) [70]. In cases where pathogens are present, iron-bounded LF (holo-LF) translocates into the cell membrane via endocytosis. The entry of LF to the cell membrane causes the release of iron radicals into the cytoplasm. The progress causes oxidative stress by ROS, resulting in cell death (Figure 2B) [70,71]. Periodically, the chelation activity of LF acts as an inhibitor on bacterial surfaces containing biofilm. Apo-LF also binds to free irons to transform into holo- or mono-LF (single-iron-bounded LF). By these complete mechanisms, the formation of LF complexes represses the viability of bacterial strains (Figure 2C) [72].

Studies on *E. coli* and *Salmonella typhimurium* (*S. typhimurium*) revealed that LF is capable of binding with LPS. The binding of LF to LPS plays a crucial role in the destabilization of the outer membrane on the bacterial surface. As a result, this activity is essential to promote the removal of glycolipids from bacterial surface or chelate Fe^+3^ ions independently [33]. In accordance with established studies, both bovine and porcine LF have been proven to diminish the growth of *Enterotoxigenic E. coli* (ETEC) strains. Therefore, the current literature indicates that their behavior as a degradative factor for virulence factors provides alternative antibiotic functions [34].

To date, numerous studies highlight the antimicrobial influence of Lf on *E. coli*, *Staphylococcus aureus*, and *Pseudomonas aeruginosa* [73], *Streptococcus mutans* [30], *Salmonella* enterica serovar Typhimurium [74], *Helicobacter pylori* [75], *Aggregatibacter actinomycetemcomitans* [31], and *Mycobacterium tuberculosis* [32]. In parallel with earlier findings, Manal M. et al. [76] primarily focused on the effect of LF on Pseudomonas aeruginosa. During the experiment, it was reported that the concentrations were lower than killing or terminating growth. By employing further investigation, it has been confirmed that LF stimulates bacteria, causing them to wander across surfaces instead of forming clusters or biofilms [69]. 

Currently, LF sources are diverse, extending beyond human and bovine origins to include camels, sheep, porcine species, and others. An extensive analysis was performed to summarize different sources of LF and the purification of camel milk. The primary objective of this research is to discuss results about alternative LF sources and various effects, such as antimicrobial activity [77]. The final result demonstrated that LF was mostly effective against methicillin-resistant *Staphylococcus aureus* (MRSA), *Staphylococcus aureus*, *K. pneumonia*, multidrug-resistant (MDR) bacteria, and *Pseudomonas aeruginosa*.

Bovine colostrum appears to be enriched by a variety of favorable components, including LF. As indicated by recent studies, the antimicrobial activity of bovine colostrum has been highlighted with many beneficial characteristics [5,78,79,80,81]. In vitro studies revealed an adhesion of ETEC bacteria to gut epithelial cells. Extensive research has reported that ETEC virulence factors also exerted an effect on this activity [82,83,84,85]. In accordance with Dierick et al. [86], the investigation fundamentally focused on these effects to determine whether both bLF and porcine LF (pLF) can mitigate ETEC-induced diarrhea in vivo or not. Experiments were conducted with bLF due to easier accessibility. Based on experimental reports, both bLF and pLF are capable of reducing the absorption of ETEC-induced fluid and bacterial adhesion to the intestinal epithelium. Correspondingly, further methodological observations reported that the absorption level demonstrated a direct ratio between both LF sources. In contrast, LF, in general, did not alter ETEC-induced innate immune genes.

### 2.2. Probiotic Effect 

LF stimulates the growth and diversification of specific probiotic strains in the intestinal microbiota [87]. In a leading research study, which includes bLF, hLF, and their derivatives, the aim is to highlight the probiotic effect of these constituents in vitro. Antimicrobial activity was also monitored against MRSA. In alignment with these findings, their interaction with intestinal microbiota is objectively identified [88,89].

LF acts as a prebiotic agent against *Bifidobacterium* (specifically on *B. infantis*, *B. bifidum*, and *B. longum*) and *Lactobacillus* species by providing necessary iron ions and *N*-glycans to interact with their cell walls to promote their growth [6,90]. The utilization activity of LF possesses a valid role to increase their gene expression [2,36,91,92,93]. Additionally, LF possesses a greater bifidogenic activity than human mature milk compared to colostrum [94]. Moreover, LF exhibits this activity via the presence of lacto-*N*-biose (a natural disaccharide naturally present in human milk), which serves as a growth promoter and metabolic activity enhancer substrate for bifidobacteria [36,95]. Based on experimental results, bLF exhibited a growth-promoting effect on *Bifidobacterium breve*, *Lactobacillus coryniformis*, *Lactobacillus delbrueckii*, and *Lactobacillus acidophilus* [37]. 

In light of evidence from recent studies, the higher growth-promoting activity of LF was found in human milk rather than cow or goat milk. The results were obtained in cultures of *Bifidobacterium infantis*, *Bifidobacterium bifidum* subsp. *pennsylvanicus* (*B. pennsylvanicus*), and *Bifidobacterium longum* [36]. Contrary to reports, growth-promoting effects on *B. infantis*, *B. breve*, and *B. pennsylvanicus* were similar in human and cow milk [36]. As a result, the inhibitory effects of human breast milk on *B. breve* and neonatal pathogens, including *E. coli* and *Staphylococcus epidermidis*, were documented [96].

Pasteurization may influence the inhibitory effects of bioactive components in milk. An analysis by Vega-Bautista [97] reported that unpasteurized milk exhibited a stronger diminishing effect on the bacterial strains, including *B. breve*. This effect was promoted by the addition of hLF and compared with bLF. These findings indicated the hindering effect of pasteurization on the antimicrobial level, as well as the blocking effect of hLF on selected probiotic strains [97]. Moreover, the growth of probiotics is influenced by a variety of factors, including time, temperature, and iron bioavailability [35,93]. BLF can serve as a blocking mechanism for pathogenic strains under both aerobic and anaerobic conditions. This characteristic mechanism occurs simultaneously with promoting the growth of selected probiotic strains [36,37]. Based on experimental reports, it has been demonstrated that bLF affects the growth of *B. infantis* and *B. breve* in a dose-dependent manner but not the growth of *B. bifidum* [98].

In accordance with established studies, there are numerous studies about apo- and holo-LF. As observed by performed trials, apo-LF exerts a restriction behavior on foodborne pathogens but not on lactobacilli and bifidobacteria. However, holo-LF stimulates the growth of lactobacilli selectively, not bifidobacteria [94,99]. Further studies revealed that metal-bound lactoferrin forms, such as copper- or zinc-bound LF (Cu-bLF or Zn-bLF), suppress pathogenic strains more effectively than apo-LF [100]. Hence, hLF and bLF are also used in studies to avoid necrotizing enterocolitis (NEC) in preterm infants [101].

Recent studies confirmed that bLF promotes the growth of *Lacticaseibacillus rhamnosus* GG (LGG) under cold conditions (approx. 22 °C) via modulating several central molecular pathways. Further analysis validated that bLF supplementation reduces the energy requirements and maintains carbon metabolism balance in LGG. This progress amplifies the survival rate and growth in cold conditions of LGG. It is also noted that these effects are dependent on the concentration levels of bLF supplementation [102]. A comparative investigation involving in vivo expression by Nicholas D et al. [103] was conducted to identify correlation between recombinant lactoferrin (rLF) and metabolic disorders in obese mice. The state of the art of this research was to search for the impact of various inactivated probiotic formulations. The clinical trials validated that inactivated probiotics possessed no effect on insulin sensitivity but did improve glucose tolerance and reduce inflammation in connective fat tissues.

### 2.3. Anti-Inflammatory Impact

The anti-inflammatory properties of LF are emerging as key factors in promoting overall health preservation and providing nutritional support to the body [104]. LF influences cell migration, interacts with LPS, and modulates ROS [104,105,106]. It exhibits both pro- and anti-inflammatory interferences by directing T-lymphocyte maturation into Th1 cells (T-helper cell type 1) or Th2 cells (T-helper cell type 2), contributing an inflammatory response [107]. Moreover, it induces the production of intferleukin-10 while reducing tumor neurosis factor alpha (THF-α). This counteraction in THF-α promotes a Th2-like anti-inflammatory response [108]. By binding to the lipid portion of LPS, LF inhibits the interaction of Toll-like receptor 4. Consequently, the initiation of the inflammatory cascade starts to exhibit anti-inflammatory cytokine production [33]. Additionally, LF reduces ROS production through its iron-binding mechanism and is able to modulate immune cell migration by altering fibroblast gene expression, contributing to the inflammatory response [109].

The anti-inflammatory effects of LF contribute to its antimicrobial properties and possess a wide range of potential benefits with supplementation in preterm infants. First of all, LF possesses the capacity to diminish infections, lessen the risk of NEC, and improve overall nutritional status [103,104]. In accordance with earlier reports, a comprehensive investigation suggested that different metal saturation levels of Lf on the intestinal barrier mechanism may influence inflammation. The findings, along with previous studies, proved that LF supplementation supports gut health and influences inflammation without additional side effects [110,111,112]. A study examined four forms of LF, namely, apo-LF, holo-LF, manganese-saturated lactoferrin (MnLF), and native lactoferrin (nLF). According to the results, apo-LF exhibited the strongest inhibitory and pro-inflammatory effect without causing any cytotoxicity in Caco-2 cells (a human epithelial cell line derived from colon carcinoma) [106]. Despite significant progress, limited attention has been given to nLF, in turn requiring further investigation.

As stated by an article published in 2023, a relationship between bLF and hLF against the side effects of therapy with antibiotics such as anti-inflammatory drugs, steroids, and psychophysical stress was reported [113]. According to results, LF reduced the effect of nonsteroidal anti-inflammatory drug (NSAID)-induced gastrointestinal injuries by 47–70%. Additionally, LF combined with NSAID exhibited a total improvement in the clinical results. In another article published in 2023, the interaction between LF and essential food components such as trace elements and polyphenols was analyzed [114]. According to results, LF strengthened with copper exhibited higher anti-inflammatory activity. The logic behind this activity is attributed to the prohibition of the production of inflammatory mediators including IL-6, IL-1ß (pro-inflammatory cytokines), TNF-α, and prostaglandin E2, naturally occurring prostaglandin with oxytocic properties, in stimulated cells. As reviewed in further data, lower copper-fortified LF increased the anti-inflammatory effect on stimulated macrophages compared to higher levels. Similarly, in another study, LF exhibited more redundant activities on IL-6 levels and bacterial activity [115].

### 2.4. Antioxidant Effect

The dietary supplementation of LF has demonstrated significant antioxidant effects [116]. The antioxidant activity of Lf appears to be linked with its ability to chelate iron ions [8]. Microglia, immune cells of the brain, contribute to the iron accumulation through immune-like functions. Increased LF synthesis can stimulate excess iron accumulation [18], in which LF is concentrated in brain areas with dopaminergic neurons, where this concentration may help to manage ion accumulation or reduce ROS-induced damage [27].

The potential antioxidant activity of LF has been extensively investigated [8,116]. A study concluded in 2008 suggested that bLF interacts with hydrophilic antioxidants such as glutathione and superoxide dismutase [40]. The antioxidant properties of LF became more prominent at a higher dosage of 200 mg per day, suggesting a dose-dependent effect. Moreover, another study reported findings on equine LF (eLF) in comparison with pLF and bLF [42]. According to results, due to the complexity of oxidation processes, a single method was not sufficient to fully evaluate the antioxidant capacity of bioactive molecules. The antioxidant activity of eLF exhibited a higher capacity at 10 mg/mL. However, the activity of eLF remained lower than that of pLF. In another investigation, interactions of dihydromyricetin (DMY) and myricetin (MY) (types of flavonoids) with bLF were analyzed [43]. The results indicated that the complex formulation of bLF with DMY and MY improved the stability and the antioxidant activity of both flavonoids. Moreover, MY demonstrated a stronger binding affinity to bLF, which significantly influences the biological activity and structure of flavonoids.

To date, research has identified various aspects of LF and its antioxidant activity [8,43,117]. A recent in vivo study found that high hydrostatic pressure (HHP) enhances LF hydrolysis, amplifying its antioxidant activity [118]. In a related case, the antioxidant activity of trans-resveratrol (Res) and pterostilbene (Pte) was compared [119]. Extensive analyses revealed that LF-Res had greater antioxidant activity compared to LF-Pte.

Current research is exploring the potential of LF as a nanoparticle carrier to improve the stability and antioxidant properties of other compounds [120]. In a recent study published in 2024, the effects and mechanisms of curcumin were discussed [121]. Trial results demonstrated that nano-micelles of LF-derived peptides improved stability, cell viability, and antioxidant activity.

### 2.5. Anticancer Effect

LF possesses the ability to modulate cytokine production in cancer by inducing apoptosis, attenuating angiogenesis, and arresting tumor growth in vitro [122]. In malignant cells, LF also blocks the transition from the G1 to the S phase in the cell cycle [26,123]. The anticancer activity of LF is closely linked to its expression, which has been observed in human kidney cell carcinomas and adjacent healthy tissue [44]. In vivo studies have explored the effect of oral administration of LF on T-cell-dependent tumors in head and neck squamous cell carcinomas [45]. The inhibitory effect of LF on carcinogenesis has gained increasing attention. An early investigation that was conducted in 2000 examined suppressive effects of bLF on chemically induced carcinogenesis in rats [46]. The trials demonstrated that bLF blocked colon carcinogenesis during the termination phase and suppressed carcinogenesis in the colon, lung, esophagus, tongue, and bladder during the post-initiation phase.

Numerous studies have reported suppressive effects of LF on carcinogenesis. In the presence of LF, this counteracting mechanism was identified on colon, lung, bladder, and mammary gland tumor cells [8,124,125]. Extensive studies performed an analysis on protease-digested lactoferrin fragments, which exhibit enhanced therapeutic properties, including anticancer activity [47]. The study used recombinant engineered LF (rthLF4) and full-length lactoferrin (flhLF). As a result, rthLF4 upregulated pro-apoptotic markers while downregulating signaling proteins involved in angiogenesis and metastasis.

In another study, the anticancer activity of LF purified from camel milk was examined [77]. Experimental results demonstrated that LF exerted dose-dependent cytotoxicity against human lung cancer cells. Furthermore, in a different study, the effect of apo-bLF and holo-bLF on HCC, which has limited treatment options, was examined [126]. As a result, holo-bLF exhibited redundant activity in early carcinogenic events and tumor burden in HCC models, acting as a chemo-preventative agent. However, apo-bLF was less effective in attenuating HCC tumors. In another clinical study, bLF and LF peptides inhibited the proliferation of liver cancer (HepG2 cells) and leukemia (Jurkat cells) [127]. Consistent with prior research, a recent study developed a novel nano-combination designed to target invasive cancer cells while sparing normal cells [128]. In this inquiry, bLF was combined with biosynthesized selenium nanoparticles (SeNPs) by using *Rhodotorula* species. As a result, the combination exhibited antiproliferative effects on various cell lines, including MCF-7, HepG-2, and Caco-2.

### 2.6. Antiviral Effect

The viral activities of LF were first demonstrated in mice infected with the polycythemia-inducing strain of the Friend virus complex (FVC) [48]. According to results, both bLF and hLF generated antiviral activity. However, bLF exhibited higher antiviral activity than hLF [129]. Several studies have been conducted on apo-LF and metal-saturated LF forms. Regarding the findings of these studies, there was no report of a significant difference in the context of activity. These apo- and metal-bounded LF forms mainly illustrated their antiviral activity in an earlier stage of infections. The mechanism of LF is constructed either by blocking cellular receptors or by direct binding to heparan sulfate glycosaminoglycans and virus particles. LF protects the host cell by hindering adhesion and entry. In summary, LF hinders the entry of viruses such as human immunodeficiency virus, respiratory syncytial virus, parainfluenza virus, and rotavirus [52,55,58,59,130].

LF is capable of deterring the growth of the herpes simplex virus. As binding to glycosaminoglycan complexes, LF blocks the entry or binds to the virus for host cells [51,131]. A study attributed to this mechanism concluded that bLF significantly blocked the viral infection of hepatitis C (HCV) virus in cultured human hepatocytes (cell type in liver tissue) by interacting directly with envelope proteins (E1 and E2 types) [53]. In opposition to HCV, studies made with bLF and HLF on human hepatitis B virus (HBV) exhibited antiviral activity only on cells. It is possible to say that during trials, samples were preincubated with HBV infection, not the virus [57]. Consequently, both hLF and bLF possessed antiviral activity on human papillomavirus (HPV) [60,132]. In another study, LF inhibited the development of erythroleukemia and viral titers by acting on immune cells responding to Feline calicivirus (FCV) [49]. To augment the activity, an early investigation examined the combination of hLF and recombinant murine interferon (rmu-IFNY) [50]. According to results, this combination improved the survival rate of FCV-infected mice.

Recent studies are gaining interest to proceed on further examinations on the antiviral activity of LF for potential therapeutic applications of reinforcements [123,133]. In an affiliated study, the activity of LF-derived peptides on colon cancer cells (HT29 and HCT8) was examined. The evidenced results confirmed that LF downregulated key points such as (p)-JAK2, (p)-STAT3, (p)-Erk, and (p)-AKT, which are involved in tumor growth and metastasis [134]. Further evidence of this study concluded that LF also suppressed the apoptosis in cancer cells and enhanced their sensitivity to antitumor drugs. Accordingly, this result was related to the tumor cells that are resistant to chemotherapy [54]. In another study, LF has also been observed on rotavirus using Caco-2/TC7 cells [135]. According to results, LF inhibited the Toll-like receptors’ (one of key innate system proteins) expression during viral infection, TLR7 specifically. Neutralization efficiency was also regulated with sialic acid content in LF. Moreover, a recent experiment reported an antiviral activity of liposomal bovine lactoferrin (LL) against human coronavirus 229E (HCoV-229E) and SARS-CoV-2 pseudoviruses [56]. Results indicated a stronger antiviral activity expression on LL compared to free lactoferrin at non-cytotoxic doses. Furthermore, its inhibitory activity on viral infection in human lung tissue cells was reported.

### 2.7. Immunomodulatory Impact

Immunomodulators are substances that can alter immune system functions by either enhancing or suppressing components of the immune response [136]. Several immunomodulators, including LF, have been studied in both the adaptive and innate immune systems. LF is able to bind iron and interact with various components of the host and pathogens [57,129]. Upon binding to LPS, LF regulates cytokine production to prevent inflammation and tissue damage [22,137,138]. As an immunomodulator, LF directly activates the immune response during bacterial infections [136]. In models of *Staphylococcus aureus* infection, LF increases pro-inflammatory cytokine TNF-α levels while decreasing interleukin-5 (IL-5) and interleukin-10 (IL-10) levels, confirming that LF accelerates the inflammatory response of host organisms to the infection [61].

LF interacts with antigen-presenting cells (APCs), such as macrophages, dendritic cells, and B-cells [136]. Moreover, macrophages play a critical role in the response of the innate immune system to infections by directly eliminating infected cells or secreting cytokines to limit pathogen replication [139,140]. LF receptors are located on the surface of macrophages, where these receptors present antigens to antigen-specific CD4+ T cells in the adaptive immune system [141,142]. Additionally, LF can increase the phagocytic activity of macrophages that are either inactive or infected. Lf also modulates the dendritic cell pathway and can restrict T-cell activation [65]. Both bLF and hLF bind to the surface of peripheral blood-derived dendritic cells, leading to the expression of LF receptors [143]. These interactions are crucial for supporting both innate and adaptive immune cells, particularly in T- and B-cell responses [16]. In the adaptive immune system, T-cell activity is critical. LF can cross-link T-cell receptors, promoting the activity of Th1 and inhibiting Th2. These actions help to regulate immune responses, such as those involved in allergic rhinitis, by balancing the expression of Th1 and Th2 cytokines. Hence, LF plays a role in regulating cells so they can mitigate inflammatory responses [66].

Numerous studies have investigated the immunomodulatory effects of LF. Based on a performed test, it appears that liposomal LF possesses the potential to be considered as an immunomodulator in vitro [62]. According to experimental results, LF liposomes inhibited the release of inflammatory molecules, including IL-8 and monocyte chemoattractant protein-1 (chemoattractant for monocytes), without causing cytotoxicity. Another study identified an immunomodulatory effect of bLF during SARS-CoV-2 infection. Their results indicate that bLF may influence the immune response during infection [63]. In this study, bLF was shown to reduce IL-6 levels in healthy peripheral blood mononuclear cell (PBMC) cultures and to increase chemokine ligand 5 (CCL5) in COVID-19 PBMC culture supernatants. Additionally, bLF enhanced the expression of interleukin-1 beta (IL-1β) and IL-6 mRNA in lung tissue.

Researchers have also compared the immunomodulatory effects of LF with other components, such as lactoferricin [64]. In a particular study, commercial and recombinant LF and LFcin were applied to mice. The results revealed that LF and LFcin produced significant anti-inflammatory effects by reducing interleukin-12 (IL-12) levels. However, recombinant LFcin exhibited stronger immunomodulatory activity compared to both commercial LF and LFcin.

### 2.8. Therapeutic Potential

It has been demonstrated that LF can possibly mediate various biological mechanisms such as inflammation, infections, and metabolic or pathogenic pathways. LF also possesses the ability to bind with regard to target sites through several investigations [144]. These findings have expanded its potential applications in theoretical studies. As previously mentioned, LF is a glycoprotein that provides many functions, and its effects are varied, such as immunomodulatory, antimicrobial, anticancer, antiviral, probiotic, and antioxidant effects. It is also capable of modulating both innate and adaptive immunity, apoptosis induction, and tumor suppression. In addition, LF has demonstrated efficiency against a range of infections and diseases, including bacterial, viral, and fungal infections. These characteristics have encouraged researchers to investigate LF as a potential therapeutic agent for the prevention and treatment of diabetes [145]. As an illustration, a research study focused on the prospects of using holo-bLF and hLF to treat HCC. During the study, it was found that hLF downregulated the HCC tumors compared to normal liver tissue. However, this result was found to be associated with low survival rates among HCC patients [127]. Holo-bLF also reduced early carcinogenic events, such as necrosis, ROS production, and the activation of facultative stem cells in a diethyl nitrosamine-induced HCC in vivo model. In another study, apo-bLF triggered apoptosis in HeLa cells through an oxidative stress mechanism, which incorporated increased ROS levels and decreased glutathione (a key metabolite in cellular functions) levels [146].

Rheumatoid arthritis patients often experience an accumulation of iron in their synovial fluid [147]. Excess iron is bound by LF to prohibit it from reacting with superoxide. This action helps to block the production of harmful hydroxyl radicals, and these radicals can cause important tissue damage [140]. According to clinical trials, LF is suggested as a prominent anti-inflammatory treatment for rheumatoid arthritis [141]. Researchers study how LF and LPS may treat microbial infections. It has been suggested that LF may help to terminate inflammation by binding to bacterial endotoxins and preventing the synthesis of pro-inflammatory cytokines. LF is translocated into the nucleus, and the nuclear factor kappa-light-chain-enhancer of activated B cells (NF-κB) activation is reduced [147]. Additionally, in vivo studies revealed that LF provides protection against skin and lung allergies [148,149]. Maintaining a balance between pro-oxidants and antioxidants is crucial for preventing oxidative stress [150]. An imbalance between free radical production and the antioxidant defense system can lead to damage to proteins, nucleic acids, and cell membranes [151,152]. As mentioned, LF possesses potent antioxidant activity due to its iron-binding properties. This behavior of LF helps to regulate the physiological balance of ROS production and protect cells from oxidative damage [153]. Consequently, bLF may be useful in human medicine as a food supplement to support immune function and antioxidant status [40,154].

Tear fluid contains antioxidants, including LF, which help to protect the corneal epithelium from chemical agents, ultraviolet irradiation, and direct airflow. According to the result, further research of LF utilization in eye disorders, such as keratoconus, was suggested [155]. Owing to the given therapeutic properties, LF holds significant potential as a treatment for a variety of diseases, including cancers, infections, and inflammatory conditions. Its diverse biological activities position LF as a valuable candidate for further clinical exploration.

## 3. Food Industry 

During the COVID-19 pandemic, the food industry has evolved significantly, incorporating functional food products such as functional chocolate and fermented algae [156,157,158]. LF has become a common ingredient in various dietary supplements, infant formulas, oral and skin care products, and food additives such as yogurt and beverages [159]. BLF is the most frequently used LF in the food industry. BLF was incorporated to infant formula under the brand name “BF-L” by the Moringa Milk Company in 1986 [21].

LF is primarily used as a nutritional additive in dairy products, especially yogurt, where it enhances microbial activity, sensory qualities, nutritional content, and bone health benefits [160,161,162]. The potential of LF-fortified yogurt is also being explored in Drosophila models for regulating body weight and inhibiting the growth of food-borne pathogens. In addition, it has demonstrated positive effects in treating a variety of health conditions, including acute gastroenteritis, iron deficiency anemia (IDA), and microcytic hypochromic anemia [163,164,165,166,167].

In addition to yogurt, the incorporation of LF into other foods, such as cheese and cream, has been considered to determine its impact on composition and shelf life. Studies have also explored the use of LF in sausages, where it was combined with carboxymethyl cellulose (CMC) to evaluate its effects on food properties [168,169,170]. Initial investigations into LF fortification in infant formula included the quantitative determination of bLF, its role in iron metabolism, and its bioavailability. These fortified formulas have been studied for their potential to address various health issues, such as diarrhea, respiratory infections, acute gastrointestinal symptoms, and anemia in low-birth-weight infants [171,172,173,174,175,176,177,178]. Moreover, apart from its current use in food products, LF is under consideration as a potential material for food packaging due to its antioxidant and antimicrobial properties [179].

### 3.1. Applications of LF in the Food Industry 

As mentioned in the previous section, LF has been used as fortification and supplementation on various foods in order to explore its attributes regarding different categories of food (Table 2). 

As indicated in the table, numerous studies have investigated LF fortification. One such study conducted last year focused on inhibiting the growth of foodborne pathogens [182]. The experiments were performed on *Bacillus cereus* (*B. cereus*), *Enterococcus faecalis* (*Ent. faecalis*), and *Candida albicans* (*C. albicans*) to evaluate the antimicrobial effects of LF. The findings demonstrated significantly improved effects against these pathogens, especially under refrigerated conditions. Among the yogurt samples, those treated with 1.5% LF exhibited the highest reduction in *B. cereus* and *C. albicans* compared to the 0.5% LF concentration.

In 2022, another study investigated the effectiveness of LF-fortified yogurt in alleviating the obesity-induced pancreatic dysfunctions in rats [164]. The results confirmed a remarkable enhancement of pancreatic function and some histological changes in the pancreas. Supplementation with LF (100 mg/kg body weight) combined with *Lactobacillus acidophilus* as a probiotic was particularly effective in improving pancreatic health. LF-fortified yogurt has also been examined for its health benefits and sensory enhancement. One study proposed investigating its role in treating IDA and microcytic hypochromic anemia in children [166]. It was established that the level of hemoglobin (Hb) and several parameters of red blood cells (RBCs) considerably improved in children who were on LF-fortified yogurt. The effect was significantly greater than that of the children who were receiving LF only.

In accordance with established studies, the fortifying applications of LF are widely distributing to expand its supplementation options [168,169,170,172,180]. As an illustration, a recent study investigated the supplementation of cheddar cheese with LF [168]. The research examined whether LF affected the composition, texture, or sensory properties of cheese. The results indicated that the fatty acid composition of cheese was not influenced by LF supplementation, and LF appeared to be safe for use in cheddar cheese. Another study focused on sausages, where LF was combined with CMC at concentrations of 5% and 10%, along with a 20% edible coating [169]. This investigation aimed to assess the antimicrobial effects and shelf life. The results were dose-dependent, with the coating showing overall improvement in shelf life. In 2022, another study examined LF-treated chicken breasts to evaluate shelf life and microbial efficacy. The results indicated that LF improved shelf life, with varying effects depending on the concentration used [170]. In another investigation, LF was incorporated to pasteurized cream made from cow’s milk. The study found that LF improved the shelf life of the cream and exhibited a dose-dependent antibacterial effect against *Pseudomonas aeruginosa*, *Staphylococcus aureus*, *E. coli*, and *S. typhimurium*. A recent study also explored the use of LF in nanoparticle form, which demonstrated an increased shelf life effect on fresh apples [185]. Despite these findings, no accurate data on the safety usage of the maximum dose for these effects remained. Consequently, extended studies are required.

LF has also been studied for its infant nutrition due to its significant presence in human milk and colostrum [81]. This has led to studies investigating the supplementation of LF in infant formulas. Among other studies, one study investigated the effect of LF-fortified infant formula on the antibody response [172]. However, the investigation reported an unexpected finding that low-iron formula-fed infants had higher levels of *Haemophilus influenzae* (Hib) IgG at 12 months, and LF supplementation exhibited no effect on the overall vaccine IgG response. It was also reported that infants who were breastfed had lower levels of vaccine IgG than those who received infant formula. A further study looked at the impact of bLF probiotic infant formula on the prevalence of respiratory and diarrhea-related infections in infants [174]. The results exhibited a significant reduction in morbidity from diarrhea and respiratory illnesses in infants with anemia. Additionally, a study on LF-fortified formula for acute gastroenteritis symptoms in children aged 12 to 32 months indicated that LF supplementation decreased the prevalence of symptoms [175].

As mentioned in previous sections, LF has multifunctional priorities, including antimicrobial activity, immune modulation, and iron-binding capacity, positioning it as a valuable ingredient in functional groups for supporting gut health and immunity. Given the increasing consumer demands for natural and clean-label products, LF’s natural origin and potential for inclusion in minimally processed food formulations ensure this growing demand. To extend the studies, the effects of LF with prebiotics and probiotics that could lead to the development of novel products to improve gut microbiome balance can be investigated [189,190]. Additionally, rising animal protein demand and scarce resources increased the necessity for feed additives. There are several studies that investigate the potential of LF to improve these effects along with bird health. However, investigations into novel feed additives to improve feed efficiency and growth performance remain limited [191].

Similarly, plant-based dairy products are on the rise due to potential health benefits and nutrient preferences. Despite their nutritional value, their nutritional profile can be enhanced when it is fortified with additives, like LF. Therefore, future research should investigate the incorporation of LF in plant-based dairy alternatives to enhance their nutritional value and appeal to more consumers, including health-conscious and vegan people [192]. In relation to plant-based diary, polyphenol-rich food groups are highly increasing the interest in consumer demands [193]. Therefore, evaluating their nutritional profile with additional studies is required. Furthermore, developing cost-effective and sustainable methods, such as combining various natural antimicrobials with food preservation methods, could significantly reduce production costs and increase accessibility [189,192].

### 3.2. Ensuring Food Safety in the Use of LF

Food safety while supplementing additional components may lead to a variety of irreversible consequences. The safety has been incorporated in a variety of aspects, including such techniques as freeze-drying to enhance food safety by preserving bioactive compounds [194]. To mitigate potential risks, tests for genotoxicity, animal toxicity, chronic toxicity, acute toxicity, and allergenicity must be performed under toxicology studies. LF has been applied in several food industries, and, during these analyses, researchers must consider potential toxicities. A paper published in 2012 by the EFSA (European Food Safety Authority) reported that the highest dose of bLF tested in sub-chronic toxicity studies was 2000 mg/kg body weight per day, and no toxicity was observed at this level [195,196].

In recent years, various studies have focused on bLF-peptide toxicity analysis. One such study, conducted in 2021, examined the dual mechanism of LF-derived peptides with angiotensin 1-converting enzyme inhibitory (ACE) and anticoagulant activities [197]. The results suggested that bLF-derived peptides could be potential food ingredients with antihypertensive and anticoagulant properties. The safety of these peptides was evaluated using ToxinPred (an in silico method), and they were predicted to be non-toxic. These findings support the necessity for further toxicity evaluations before any potential applications.

Similarly, a 2018 study focused on the antiamoebic activity of synthetic bLF-derived peptides, including lactoferrampin (LFampin) and LFcin [198]. According to results, LFampin demonstrated amoebicidal effects without significant toxicity. However, it was observed that LFampin primarily induced necrosis rather than apoptosis in trophozoites. Although LF-derived peptides showed promise as safer alternatives, the research emphasized the importance of further cytotoxicity examinations before they are widely applied.

These studies demonstrate no significant toxicity; however, long-term studies on the safety of dietary LF remain limited. Thus, findings of studies such as beneficial effects like improvement on immune response and gut health need further investigations to confirm these findings over extended studies. Moreover, the potential for cumulative effects such as altered gut microbiota composition or immune modulation or any other adverse effects requires further investigation to address safety questions like immunogenicity and immunotoxicity potential [196,199]. Currently, there is no maximum safe dose of dietary LF, especially for prolonged use in children and the elderly. Therefore, expanding the long-term safety of LF with evidence and filling the gaps are needed over extended studies [192,199]. Additionally, further investigations are required to meet regulatory requirements for the use of lactoferrin in fortified foods across different countries, particularly in regions with strict food safety regulations [192].

## 4. Conclusions

Investigations conducted on various sources of LF have generated considerable interest in examining its behavior under diverse conditions and applications. Antimicrobial, antioxidant, antiviral, anti-inflammatory, anticancer, prebiotic, and probiotic effects of LF have been widely studied and demonstrated to possess substantial therapeutic potential. As mentioned, LF has the ability to chelate iron to suppress the growth of various infections, a property that contributes to its rich antimicrobial activity. This activity also gives LF its anti-inflammatory and antioxidant effects. Previous studies have demonstrated that LF exhibits antioxidant activity by preventing ROS damage. Additionally, the bifidogenic activity of LF, particularly towards *B. infantis*, *B. bifidum*, and *B. longum*, supports its high probiotic potential. Despite the effect of LF on various microorganisms, diseases, gut microbiota, and immune modulation, maximum dosage for usage for safety remains unknown. Correspondingly, further investigations are required to determine more accurate standard values for a safety usage.

In recent years, the therapeutic applications of LF have advanced significantly. Researchers have been searching for LF, its derivatives, and its subsequent medicinal properties, such as its anticancer properties. As previously and comprehensively described, LF has also been included in the food industry. The fortification of LF has expanded its applications, particularly as an antimicrobial and antioxidant agent. Given the increasing trends and consumer demands of its application into the food industry, the necessity to comprehend its toxicity levels should be made a priority. Tests carried out for toxicity assessment should be directed towards genotoxicity, chronic toxicity, allergy testing, and standard thresholds should be determined. Additionally, extended studies about long-term effects of dietary LF to fill the gaps in and enhance the nutritional profile is required.

Despite established findings, certain aspects regarding the use of LF remain relatively unexplored. Therefore, numerous revisions concerning newer supplements and drugs are necessary, particularly in the development of biological agents for the food industry. Correspondingly, LF fortification on sustainable and functional food groups should be extended to reach out to larger consumer demands. These studies can enhance the nutritional profile of these groups, especially when they are conducted on trending demands such as plant-based dairy products or phenolic-rich food groups. It is crucial to begin every subsequent preclinical trial with a clear focus on toxicity parameters. Innovations in toxicity studies of LF designed for fortifying foods will contribute further implications in the future. LF, due to its multifunctional properties, has the potential for advancement in a multitude of therapeutic applications. The nutritional profile of LF can be extended through larger populations by enhancing cost-effective and more efficient methods to regulate food safety in many countries, significantly for some regions with stringent food regulations. This review mainly underscores the requirement for additional research on LF to comprehensively understand its unique traits and to explore and develop innovative applications in the food industry.

## Figures and Tables

**Table 1:** Studied fields on lactoferrin regarding its properties.

Effect	Lactoferrin Source	Function	References
Antimicrobial effect	bLF from cheese whey protein	Enhance antimicrobial effects against *Escherichia coli*, *Staphylococcus aureus*, *Coagulase negative-staphylococci (CNS)*, *Pseudomonas aeruginosa*, and *Klebsiella pneumoniae*.	[29]
	hLF	Exhibit protection against *Streptococcus* mutants.	[30]
	hLF	Increase defensive effects on *Aggregatibacter actinomycetemcomitans*.	[31]
	bLF	Protect against *Mycobacterium tuberculosis*.	[32]
	bLF and hLF	Disrupt the bacterial cell wall. Interact with lipopolysaccharides.	[33]
	bLF and porcine LF	Decrease the growth of enterotoxigenic *Escherichia coli*.	[34]
Probiotic effect	bLF	Decrease necrotizing enterocolitis.	[35]
	hLF	Enhance growth-promoting activity on *Lacotbacillus* and *Bifidobacterium* species, especially on *Bifidobacterium infantis*, *Bifidobacterium bifidum*, and *Bifidobacterium longum*.	[36]
	bLF	Enhance dose-dependent suppressing effect on *Bifidobacterium breve*, *Lactobacillus coryniformis*, *Lactobacillus delbrueckii*, and *Lactobacillus acidophilus*.	[37]
Prebiotic effect	bLF	Decrease the growth of *Bifidobacterium breve*, *Bifidobacterium angulatum*, *B. catenulatum*, *Bifidobacterium bifidum*, *Lactobacillus coryniformis*, *Lactobacillus delbrueckii*, *Lactobacillus acidophilus*, *Lactobacillus reuteri*, and *Lactobacillus paraplantarum*. Increase the growth of *Lactobacillus rhamnosus*, *Lactobacillus paraacasei*, and *Pediococcus pentosaceus*.	[38]
Gut microbiome eubiosis	bLF	Ensure retention of microbiome diversity. Inhibit the formation of harmful microorganisms in the microbiome in pediatric patients. Modulate abundance of beneficial bacteria in pediatric patients.	[39]
Antioxidant activity	bLF	Increase total T cell. Increase antioxidant capacity.	[40]
	LF (purified from cow milk)	Increase antioxidant activity. Protect iron and immunodeficiency. Decrease the risk of sepsis in premature infants with very low birth weight. Ensure retention of beneficial gut microbiota.	[41]
	eLF	Increase antioxidant activity.	[42]
	bLF	Protect the stability and antioxidant activity of both dihydromyricetin and myricetin. High binding affinity of myricetin to bLF.	[43]
Anticancer effect	LF	Increase positive immunoreaction in human sporadic renal cell carcinoma.	[44]
	hLF	Decrease T cell-dependent tumor of head and neck squamous cell carcinoma. Inhibit tumor growth.	[45]
	LF	Inhibit developed colon, lung, bladder, and mammary gland tumor cells.	[8]
	bLF	Inhibit colon carcinogenesis in termination phase. Inhibit colon, lung, esophagus, tongue, and bladder carcinogenesis in post-initiation phase.	[46]
	Recombinant engineered LF	Enhance pro-apoptosis markers. Decrease the signaling proteins of angiogenesis and metastasis.	[47]
Antiviral effect	hLF and bLF	Increase protective effects of Friend virus complex.	[48]
	hLF and bLF	Inhibit Friend virus complex.	[49]
	hLF and recombinant murine interferon	Enhance antiviral activity on polycythemia-inducing strain of the Friend virus complex.	[50]
	LF	Inhibit herpes simplex virus (HSV).	[51]
	LF	Inhibit human immunodeficiency virus (HIV).	[52]
	LF	Prevent the growth of hepatitis C virus (HCV).	[53]
	LF-derived peptides	Increase anticancer activity on colon cancer cells.	[54]
	LF	Inhibit rotavirus infection.	[55]
	Liposomal bLF	Exhibit antiviral activity against HCoV-229E and severe-acute-respiratory-syndrome coronavirus-2 (SARS-CoV-2).	[56]
	hLF and bLF	Increase antiviral activity against hepatitis B virus.	[57]
	LF	Exhibit antiviral and antibacterial activity on respiratory syncytial virus. Increase antiviral and antibacterial activity on cytomegalovirus.	[58]
	LF	Decrease the growth of parainfluenza virus.	[59]
	hLF and bLF	Inhibit human papillomavirus (HPV).	[60]
Immune-modulatory effect	LF	Exhibit immune modulator effect on *Staphylococcus aureus*-infected models.	[61]
	Liposomal LF	Decrease the release of inflammatory molecules including interleukin-8 (IL-8) and monocyte chemoattractant protein-1 (MCP-1).	[62]
	bLF	Exhibit immunomodulatory effect on SARS-CoV-2.	[63]
	LF and LF-derived peptides (Lactoferricin)	Exhibit immunomodulator activity.	[64]
	LF	Increase the T-cell activation.	[65]
	LF	Inhibit allergic rhinitis. Regulate anti- and pro-oxidant cell expression.	[66]

**Table 2:** Functions and properties of LF usage in foods.

Food	Concept	Textural and Sensory Properties	Function	References
Yogurt	LF and chia seed mulcilage	Exhibit superior assembly in the yogurt matrix.	Enhance intestinal delivery. Ensure retention of antioxidant activity.	[180]
	LF-fortified yogurt	Increase sensory properties.	Exhibit significant antimicrobial activity against *E. coli* and *S.typhimurium*. The resistance of *Pseudomonas aeruginosa* and *Bacillus cereus*.	[181]
	LF-fortified yogurt	*	Increase antimicrobial effect against *Bacillus cereus*, *Ent. faecalis*, and *Candida albicans*.	[182]
	LF-fortified yogurt	*	Exhibit high efficiency in body mass reduction. Decrease glycated hemoglobin ratio.	[183]
	LF-fortified yogurt, using YC-X11 yoghurt	No significant change in the structure of yogurt.	Improve shelf life.	[163]
	LF-supplemented stirred yogurt	Improve liquid profile.	Increase pancreatic function. Enhance histological change in the pancreas.	[164]
	LF-fortified yogurt	Exhibit firmness with acceptable sensory traits.	Increase hemoglobin levels.	[165]
	LF- fortified yogurt	*	Decrease the symptoms of the disease.	[166]
	BLF dosed at up to 0.15% (*w*/*w*) into stirred yogurt	- No apparent effect on some physical and sensory properties of yogurt. - Conserve composition for at least 21 days at 4 °C.	Exhibit osteogenic activity in bone-forming cell cultures.	[167]
Cream	LF- fortified cream	*	Improve shelf life. Exhibit antibacterial effect against *Pseudomonas aeruginosa*, *Staphylococcus aureus*, *E. coli*, and *S. typhimurium*.	[184]
Cheese	LF-supplemented cheddar cheese	Protect color, flavor, or texture score.	Sustain fatty acids composition.	[168]
Sausage	LF-fortified homemade sausage with CMC and edible coating	*	Exhibit dose-dependent antimicrobial effect. Improve sausage quality and shelf-life by edible coating.	[169]
Chicken breast	LF-treated chicken breast	- Exhibit dose-dependent difference between the scores of sensory properties. - Exhibit better sensory properties in 20 mg/g lactoferrin treated group. - No significant difference in taste scores.	Enhance shelf life. Exhibit dose-dependent antimicrobial activity.	[170]
Fresh apple	LF, methylcellulose, and chitosan-treated silver-based nanoparticles	*	Improve shelf life and preservation.	[185]
Formula	BLF-treated infant formula	*	No affect in vaccine immunoglobulin-G (IgG) response.	[172]
	BLF-treated infant formula	*	Degrade sepsis-causing organisms.	[173]
	BLF-fortified formula	*	Decrease diarrhea morbidity. Decrease respiratory infections.	[174]
	LF-fortified formula	*	Decrease acute gastroenteritis symptoms.	[175]
	LF-fortified formula and breast milk	*	Decrease late-onset sepsis. No net change in risk of NEC or morbidity.	[176]
	Iron-fortified formula with BLF	*	Increase hemoglobin levels of anemic infants.	[177]
	LF-fortified infant formula with bovine milk fat globule membrane	*	Enhance neurodevelopmental profile language learning of infants. Exhibit age-dependent growth of infants. Decrease diarrhea and respiratory-associated infections.	[178]
	LF-fortified liquid formula	*	Increase activity of reconstituted and IMF-supplemented LF. Increase activity of high-pressure-processing-treated LF. Increase functional traits in high-pressure-processing-treated LF.	[171]
	LF-iron-fortified milk formula	No net difference between physicochemical forms.	No net change in iron levels.	[186]
	LF-supplemented formula	*	No net change in iron transport pathway or shelf life.	[187]
	bLF-fortified formula	*	Increase LF activity.	[188]

* Not determined.
